# Neurons Help Bridge the Brain's Communication Gap

**DOI:** 10.1371/journal.pbio.1000231

**Published:** 2009-10-27

**Authors:** Rachel Jones

**Affiliations:** Freelance Science Writer and Editor, Welwyn, Hertfordshire, United Kingdom

**Figure pbio-1000231-g001:**
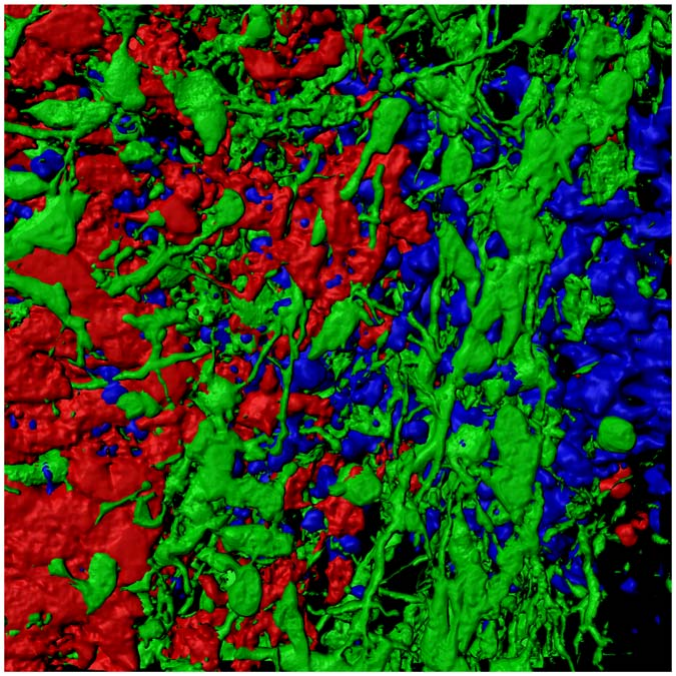
This illustration of a coronal corpus callosum section of a mouse embryo shows glutamatergic neurons (red), GABAergic interneurons (green), and other remaining cells (their nuclei in blue), which together form a complex network in which callosal axons are hypothesized to grow preferentially. Illustration: Mathieu Niquille, University of Lausanne, Switzerland.

The conventional wisdom that classifies linear, analytical thinkers as left-brained and intuitive, artistic types as right-brained is firmly rooted in the physical and functional anatomy of the brain. The cerebral cortex, which is responsible for higher-order functions like processing environmental stimuli and language, is divided into left and right hemispheres. Although each hemisphere has its own responsibilities, with the left, for example, controlling movement on the right side of the body and vice versa, it is vital that the two halves of the brain can communicate in order to coordinate their activities. Much of this communication occurs through a structure called the corpus callosum—a tract of white matter through which millions of axons cross the interhemispheric space.[Fig pbio-1000231-g001]

In a new study, Lebrand and colleagues investigate how these axons find their way from one hemisphere to the other, thereby forming the corpus callosum. Previous work has shown that a group of glia (non-neuronal brain cells) found at the midline of the brain—in particular, in a bridge-like structure known as the “glial sling”—are essential for this process. The recent finding that the glial sling contains neurons, as well as glial cells, led Lebrand and colleagues to investigate whether these neurons also contribute to axonal guidance in the developing corpus callosum.

The authors found that, in fact, two groups of neurons appear in the entire interhemispheric commissure during the period just before the arrival of the axons that will form the corpus callosum. The two types of neurons can be distinguished by the expression of different neurotransmitters; one group expresses the inhibitory transmitter GABA (GABAergic), whereas the other expresses the excitatory transmitter glutamate (glutamatergic). The GABAergic and glutamatergic neurons migrate to the developing corpus callosum from different sites within the brain and are found at the midline only transiently, disappearing later in development. Structurally, these neurons form a dense network that surrounds axons as they pass through the corpus callosum, bringing the axons and neurons into close contact with each other.

To test whether this neuronal structure is required for the normal formation of the corpus callosum, the authors examined mice lacking a gene called *Mash1*, whose GABAergic neurons fail to develop normally. These mice had almost no GABAergic neurons in the developing corpus callosum (although the glial population appeared normal), and few, if any, axons were able to cross the midline. The glutamatergic neurons at the midline were also affected, with many being displaced ventrally, away from their normal position. These findings imply that the transient GABAergic neurons found at the midline are essential for the normal development of the corpus callosum. To confirm this, the authors transplanted part of the dorsal cortex (where axons of the corpus callosum originate) from wild-type mice into cultured brain slices from *Mash1-*mutant mice and found that the wild-type axons failed to cross the midline. By contrast, when dorsal cortex was transplanted from *Mash1-*mutant mice into wild-type brain slices, the mutant axons passed normally into the corpus callosum. This supported the idea that GABAergic neurons at the midline are crucial for axon guidance in the developing corpus callosum.

Next, the authors addressed the question of how these two populations of neurons might be involved in axonal guidance. Experiments in which pieces (explants) of developing corpus callosum containing the neurons in question were placed close to a piece of cortex showed that the corpus callosum could attract growing axons toward itself, indicating that it was releasing one or more chemoattractant substances. Further coculture experiments, using explants containing or lacking either neuronal population, showed that both the GABAergic and glutamatergic neurons exert a chemoattractant effect on developing callosal axons. What consititues this chemoattractant signal? One candidate is a molecule called Semaphorin3C (Sema3C), a known axon-guidance factor that is expressed by the transient population of glutamatergic neurons in the developing corpus callosum, but not in the GABAergic neurons or the glia. In mice in which the gene for Sema3C had been inactivated, the number of axons crossing the midline was severely reduced, showing that Sema3C contributes to the formation of the corpus callosum. Coexplant experiments reveal that chemoattraction of callosal axons toward the developing corpus callosum rely, in part, on the Sema3C. This attraction is mediated through the semaphorin receptor Neuropilin 1, as shown by the ability of blocking antibodies against Neuropilin 1 or small inhibitory RNA molecules that silence expression of this receptor to block the axonal response.

These findings bring a new level of complexity to our understanding of how the corpus callosum is formed. The involvement of two previously uncharacterized populations of neurons in axon guidance across the midline raises the question of how neurons and glia interact to mediate callosal development and will surely stimulate future investigations. In particular, the identification of a further chemoattractant factor or factors, released by both neuronal populations, is likely to be a priority, as is investigation of how the callosal neurons and glia come together to form the dense structure through which axons are guided across the midline.

**Niquille M, Garel S, Mann F, Hornung J-P, Otsmane B, et al. (2009) Transient Neuronal Populations Are Required to Guide Callosal Axons: A Role for Semaphorin3c. doi:10.1371/journal.pbio.1000230**

